# Impact of Medical‐Educational Collaboration on School‐Based Mental Health Support in Japan

**DOI:** 10.1111/ped.70588

**Published:** 2026-10-29

**Authors:** Kei Ohashi, Mayu Mitani, Motoki Kurumai, Rie Yamada, Ryo Tsujino, Takahiro Takano, Masatsugu Sakata, Yukiyo Nagai, Atsurou Yamada

**Affiliations:** ^1^ Department of Neurodevelopmental Medicine Nagoya City University Graduate School of Medical Sciences Nagoya Japan

**Keywords:** interdisciplinary communication, medical‐educational collaboration, professional development, school counselor, school‐based mental health

## Abstract

**Background:**

In Japan, school‐based mental health support is becoming increasingly critical, owing to rising rates of school non‐attendance and suicide among students. However, barriers in accessing medical facilities remain high. In Nagoya, the “Children's Advocacy Committee” has implemented a medical‐educational collaboration model. This study evaluated the impact of physician participation in school case review meetings and observation of outpatient consultations on the support skills of school‐based professionals.

**Methods:**

An anonymous self‐administered questionnaire was distributed to 178 members of the Children's Advocacy Committee who participated in the collaboration program in fiscal year 2025. The participants rated the degree of change in their interactions with students, parents, and school and medical staff using a 5‐point Likert scale.

**Results:**

A high percentage of the 155 respondents reported positive changes across all categories. In the case review meetings, 68% perceived positive changes in support approaches toward students. In outpatient observations, 72% perceived positive changes in support approaches toward parents. Spearman's rank correlation analysis revealed significant negative correlations between years of professional experience and the degree of perceived change, particularly for outpatient observations (e.g., students: *ρ* = −0.395, *p* < 0.01). This indicates that staff members with fewer years of experience perceived greater benefits from the program.

**Conclusions:**

Both collaborative activities were associated with perceived positive changes in support approaches among school‐based professionals. The clinical exposure provided by outpatient observations was particularly impactful for early‐career staff. Establishing a tiered collaborative system tailored to professional experience can further strengthen school‐based mental health support.

## 1 Introduction

In Japan, the number of elementary and junior high school students who refuse to attend school or who commit suicide has increased over the past few decades, and the importance of mental health support has increased substantially. It is often difficult for elementary and junior high school students struggling with mental health issues to seek treatment in medical facilities [1]. Therefore, school counselors (SCs) and others must provide support to these students in educational settings.

In Nagoya, one of Japan's major cities, full‐time SCs have been assigned to all public junior high schools since 2014 to provide support for elementary and junior high school students facing mental health problems. The SCs have been working alongside school social workers (SSWs), school police officers (SPs), and school secretaries (SSs) as part of the “Children's Advocacy Committee” to comprehensively address mental health issues among elementary and junior high school students. Since its establishment, this system has been associated with gradual improvements in children's access to consultation services and increased satisfaction with support services [2].

This was a pioneering Japanese initiative. However, the mental health issues faced by elementary and junior high school students are becoming increasingly complex, and members of the Children's Advocacy Committee, who provide support to these students, often struggle to address them. Therefore, since 2024, pediatricians or psychiatrists have participated in team meetings of the Children's Advocacy Committee at schools to provide advisory services. Members of the Children's Advocacy Committee have attended outpatient consultations at hospitals to observe and assist in enhancing support capabilities since 2025.

This study aimed to clarify how these initiatives influenced the day‐to‐day support provided by members of the Children's Advocacy Committee. This enables the development of more effective initiatives to enhance support capabilities.

## 2 Methods

### 2.1 Data Source

We used secondary data from an online, anonymous, and self‐administered questionnaire survey of 178 members of the Children's Advocacy Committee from April 2025 to March 2026 (111 SCs, 27 SSWs, 16 SPs, 16 SSs, and eight helping professionals) for program evaluation purposes.

### 2.2 Settings

The Children's Advocacy Committee, comprising multidisciplinary professionals, including SCs, SSWs, SPs, SSs, and Helping Professionals, is divided into 17 blocks in Nagoya City. Team meetings are held approximately weekly by the members of each block. Pediatricians or psychiatrists provide guidance at these team meetings twice a year in each block (approximately 2 h per session), primarily by offering advice from a medical perspective on cases where staff struggle to find appropriate responses. During outpatient consultation observations, members of the Children's Advocacy Committee attended initial consultations of newly presenting patients who were not under their direct care (approximately 2–3 h per observation session) after obtaining informed consent from both the patients and their parents. This was done at the Research Center for Neurodevelopmental Disorders at Nagoya City University Hospital, a tertiary referral center integrating pediatric neurodevelopmental medicine and child and adolescent psychiatry. Between April 1, 2025, and March 31, 2026, 82 members participated in these consultation observations and subsequently exchanged views with the attending physicians.

### 2.3 Survey Items

The survey collected demographic and professional background data, including participants' occupations (e.g., SC, SSW, SP, SS, and Helping Professional) and years of professional experience. The primary focus of the evaluation was professional empowerment and capacity building (“supporting the supporters”). The survey evaluated the perceived changes in participants' support capabilities, clinical understanding, and confidence when interacting with four key groups: [1] children and students, [2] parents, [3] school staff, and [4] hospital medical staff. Responses were recorded on a 5‐point Likert scale (1 = no change, 3 = some change, 5 = significant change), capturing the extent to which the participants perceived positive shifts in their professional support approaches and communication strategies in daily practice.

### 2.4 Statistical Analyses

To examine the relationship between years of professional experience and the perceived degree of change, Spearman's rank correlation coefficients (*ρ*) were calculated. Bonferroni corrections were applied to adjust significance thresholds (*α*′ = 0.05/4 = 0.0125 per activity). All analyses were performed using SPSS version 29.0 (IBM Corp., Armonk, NY, USA), and correlation coefficients were calculated.

### 2.5 Ethical Considerations

This study was conducted in accordance with the principles of the Declaration of Helsinki. The analysis used secondary data originally collected for administrative program evaluation. The ethics committee of the Nagoya City University Graduate School of Medical Sciences was consulted, and the study was exempted from formal Institutional Review Board (IRB) approval and informed consent due to its non‐interventional nature and the use of de‐identified secondary data.

## 3 Results

Responses were received from 155 participants (87% response rate). Table 1 shows the number of participants according to their occupation, years of experience, and response rates. A complete summary of basic descriptive statistics for the 5‐point Likert scale items, including response counts, percentages for each scale score, means, standard deviations, medians, and interquartile ranges across all categories, is provided in Table 2. Overall, positive changes were high across all items (Figure 1). Regarding participation in case review meetings, the proportion of respondents who perceived positive changes in their support approaches was highest toward “children and students” at 68% (total of scales 3–5; the same applies below), followed by “parents” at 59%, “school staff such as teachers” at 54%, and “medical staff in hospitals” at 51%. Regarding observation of outpatient consultations, the percentage of participants who perceived positive changes in their support approaches was 72% toward “parents,” and 71% toward “children and students,” 57% toward “medical staff in hospitals,” and 49% toward “school staff such as teachers.”

**TABLE 1:** Characteristics of participants by occupation and years of experience.

	*N* (response rate, %)	Years of experience, median (IQR)
Total	155 (87)	5 (3–8)
School counselors	100 (90)	7 (4–9)
School social worker	22 (100)	3 (2–8)
School police officer	11 (69)	2 (2–3)
School secretary	16 (100)	4 (3–5)
Helping professional	6 (75)	4 (2–6)

*Note:* This table presents the number of respondents, response rates, and years of experience in each occupation within the Children's Advocacy Committee. Data for years of experience are presented as median and interquartile range (IQR).

**TABLE 2:** Detailed descriptive statistics of perceived degree of change across collaborative activities.

	1: No change, *n* (%)	2: *n* (%)	3: Some change, *n* (%)	4: *n* (%)	5: Significant change, *n* (%)	Perceived change rates (3–5) (%)	Median (IQR)
Participation in case review meetings							
Students	26 (17)	24 (15)	70 (45)	28 (18)	7 (5)	67.7	3 (2–3)
Parents	36 (23)	28 (18)	60 (39)	23 (15)	8 (5)	58.7	3 (2–3)
School staff	43 (28)	29 (19)	60 (39)	17 (11)	6 (4)	53.5	3 (1–3)
Medical staff	54 (35)	23 (15)	51 (33)	12 (8)	15 (10)	50.3	3 (1–3)
Observation during outpatient consultations							
Students	15 (16)	12 (13)	42 (45)	16 (17)	8 (9)	71.0	3 (2–4)
Parents	17 (18)	9 (10)	44 (48)	15 (16)	7 (8)	71.7	3 (2–3)
School staff	26 (29)	19 (21)	29 (32)	13 (14)	3 (3)	38.9	2.5 (1–3)
Medical staff	29 (32)	11 (12)	34 (37)	11 (12)	7 (8)	62.0	3 (1–3)

*Note:* Data are presented as counts (*n*) and percentages (%) for each response category on a 5‐point Likert scale (1 = no change, 3 = some change, and 5 = significant change). Perceived change rates indicate the proportion of respondents who reported scale scores of 3–5. Summary metrics are presented as medians (interquartile range, IQR).

**FIGURE 1:**
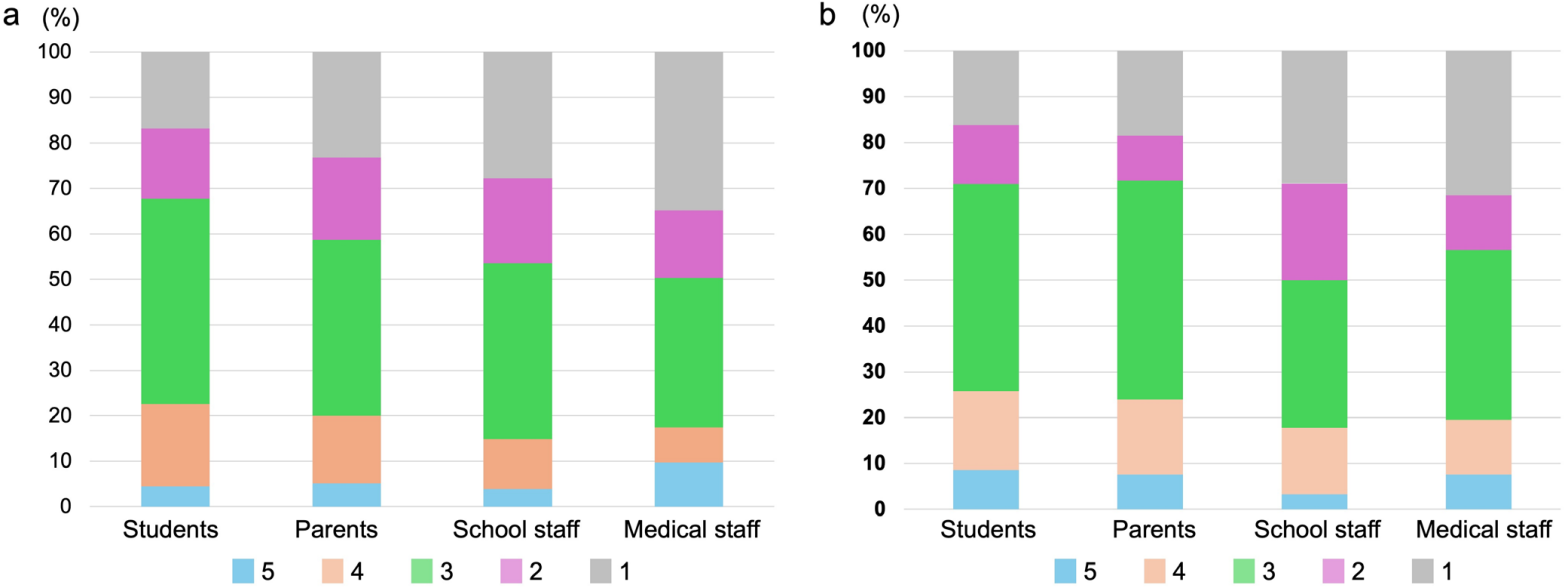
Degree of change in interactions through medical‐educational collaboration activities. The stacked bar charts indicate the degree of change perceived by the Children's Advocacy Committee members in their interactions with “students,” “parents,” “school staff,” and “medical staff.” Changes were measured on a 5‐point Likert scale (1 = no change, 3 = some change, and 5 = significant change). (a) Participation in case review meetings. (b) Observation during outpatient consultations. Data are presented as percentages (%) of all respondents.

Furthermore, analysis of the relationship between years of professional experience revealed significant negative correlations with the degree of change in several areas (Figure 2). Regarding participation in case review meetings, the weak negative correlation observed for perceived changes in approaches toward students (*ρ* = −0.165, *p* < 0.05) did not retain statistical significance after Bonferroni correction for multiple comparisons (*α*′ = 0.0125). Similarly, for outpatient observations, the correlation for hospital medical staff (*ρ* = −0.213, *p* < 0.05) lost statistical significance after correction. In contrast, robust negative correlations remained statistically significant after Bonferroni correction across key interaction categories: students (*ρ* = −0.395, *p* < 0.0125), parents (*ρ* = −0.332, *p* < 0.0125), and school staff (*ρ* = −0.306, *p* < 0.0125). These results indicate that participants with fewer years of experience tended to perceive greater positive changes through direct outpatient observations, particularly in their approaches to children, parents, and school personnel.

**FIGURE 2:**
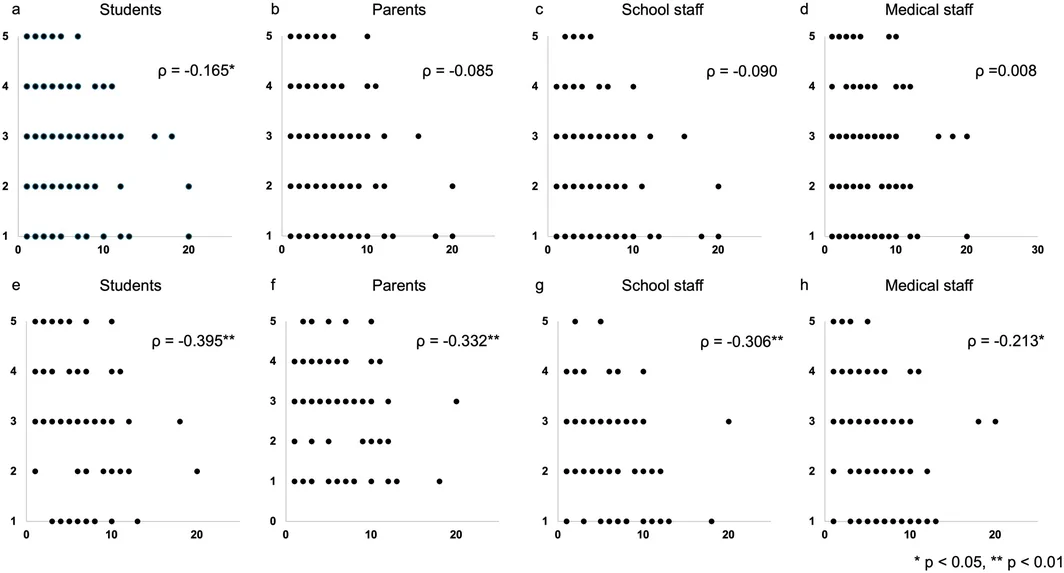
Correlations between years of professional experience and the degree of change through medical‐educational collaboration activities. The dot plots illustrate the relationship between the years of experience of the Children's advocacy committee members (x‐axis) and their perceived degree of change in interactions with various stakeholders (*y*‐axis; 5‐point Likert scale). (a–d) Participation in school‐based case review meetings: (a) students, (b) parents, (c) school staff, and (d) medical staff. (e–h) Observation of outpatient consultations with (e) students, (f) parents, (g) school staff, and (h) medical staff. Spearman's rank correlation coefficients (*ρ*) are shown for each plot. Statistical significance is indicated by asterisks: *p < 0.05, **p < 0.01.

## 4 Discussion

A central finding of this study is that school‐based professionals perceived positive changes in their own support approach and capabilities reflecting the core initiative objective often summarized as “supporting the supporters.” Rather than directly assessing clinical symptom reduction in students or objective skill acquisition, this study evaluated the extent to which professionals perceived changes in their daily interactions with students, parents, and school and hospital medical staff. Through participation in school‐based case review meetings and outpatient consultation observations, staff members gained clinical insights into pediatric mental health, which were associated with perceived positive shifts in their approaches to interacting with children, families, and interdisciplinary colleagues. In school‐based mental health systems, fostering opportunities for frontline professionals to gain medical insights and re‐examine their support approaches represents an important foundation for providing sustained support to vulnerable children.

The importance of multidisciplinary collaboration in school mental health has been widely recognized. School‐based mental health services integrating educational and medical perspectives have been shown to facilitate early identification, appropriate intervention, and improved family support [3]. Additionally, collaborative models in urban settings have demonstrated that partnerships between schools, healthcare providers, and community resources enhance the effectiveness of mental health support [4]. Our findings are consistent with this body of literature and further support the importance of incorporating medical perspectives into school‐based support systems.

Pediatricians or psychiatrists' participation in case review meetings was most strongly associated with perceived positive shifts in professionals' support approaches toward students. This finding aligns with the framework of consultation–liaison models in which expert consultation enhances case formulation and supports more effective decision‐making by frontline staff [5]. Input from medical professionals may have facilitated a deeper understanding of developmental characteristics and psychiatric symptoms, leading to more appropriate and individualized responses in school settings.

Contrastingly, outpatient consultations were associated with perceived changes in professionals' support approaches toward both students and parents. Direct exposure to real clinical encounters likely provided the participants with practical insights into the communication strategies and relationship‐building processes used by physicians. From the perspective of the social learning theory, observing the behavior of experienced clinicians can promote the acquisition of new skills and behavioral patterns [6]. Furthermore, the importance of family involvement in child mental healthcare has been emphasized in previous studies [7, 8], supporting the interpretation that outpatient observation may be especially helpful in developing professionals' approaches toward parents.

Another key finding was the inverse relationship between professional experience and perceived change. After adjusting for multiple comparisons, significant negative correlations remained, specifically for outpatient observations (students, parents, and school staff), whereas the correlation for case review meetings was no longer significant. This indicates that direct clinical exposure during outpatient observations was particularly impactful for less‐experienced staff, aligning with the educational theory that novices are more receptive to new perspectives [9]. These results highlight the utility of tailoring collaborative training to professional experience.

Future research should incorporate objective measures of practice change and examine the impact on student mental health outcomes through longitudinal designs. Additionally, identifying the key components of effective collaboration, such as frequency, intensity, and bidirectional engagement, is important for developing sustainable and scalable models of medical–educational partnerships.

This study had some limitations. First, the findings were based on self‐reported measures and did not directly assess actual behavioral changes or student outcomes. Second, this study was conducted in a single urban area in Japan, which may limit its generalizability.

In conclusion, participation in case review meetings and observing outpatient consultations were associated with perceived positive change in support approaches across a wide range of areas, including interactions with children, students, their parents, and school and medical staff. Moving forward, establishing a tiered medical‐educational collaboration system tailored to staff experience may offer a valuable framework for supporting frontline professionals.

## Author Contributions

K.O. had full access to all data in the study and takes responsibility for its integrity and the accuracy of the data analysis. K.O. conceived the study and drafted the original manuscript. M.M., M.K., R.Y., R.T., T.T., M.S., Y.N., and A.Y. critically revised the manuscript for important intellectual content. All authors have approved the final manuscript and agreed to be accountable for all aspects of the study.

## Funding

The authors have nothing to report.

## Conflicts of Interest

All authors are members of the Department of Neurodevelopmental Medicine, Nagoya City University Graduate School of Medical Sciences, which receives funding from the City of Nagoya. The funder had no role in the study design, data collection and analysis, or writing of the manuscript. Masatsugu Sakata received a personal fee from Daiichi‐Sankyo outside the submitted work.

## Data Availability

The datasets generated and/or analyzed during the current study are not publicly available due to administrative agreements and the need to protect the privacy of the municipal program participants, but are available from the corresponding author upon reasonable request and with the permission of the relevant municipal authorities.
